# Mitral valve surgery in acute infective endocarditis: long-term outcomes of mitral valve repair versus replacement

**DOI:** 10.2459/JCM.0000000000001544

**Published:** 2023-08-02

**Authors:** Lorenzo Di Bacco, Michele D’Alonzo, Massimiliano Di Mauro, Rocco Davide Petruccelli, Massimo Baudo, Camila Mayorga Palacios, Stefano Benussi, Claudio Muneretto, Fabrizio Rosati

**Affiliations:** a Cardiac Surgery Unit, Cardio-Thoracic Department, University of Brescia, Brescia, Italy; b Center for Neuroscience, Queen's University, Kingston, Ontario, Canada

**Keywords:** infective endocarditis, mitral valve repair, mitral valve replacement, valve surgery

## Abstract

**Aims:**

Timing and surgical strategies in acute infective endocarditis are still questionable. We sought to investigate clinical outcomes of patients undergoing mitral valve repair (MVR) compared with mitral valve replacement [mitral valve prosthesis (MVP)] for acute infective endocarditis.

**Methods:**

From 2004 to 2019, 109 consecutive patients with acute mitral valve infective endocarditis were retrospectively investigated. Patients were divided into two groups according to surgical strategy: MVR 53/109 (48.6%) versus MVP 56/109 (51.4%). Primary end points were in-hospital mortality and overall survival at 10 years. Secondary end point was the freedom from infective endocarditis relapse.

**Results:**

Our institutional surgical approach for infective endocarditis allowed us to achieve MVR in 48.6% of patients. Hospital mortality was comparable between the two groups [MVR: 1/53 (1.9%) versus MVP: 2/56 (3.6%), *P* = 1.000]. Overall 10-year survival was 80.0 ± 14.1 and 77.2 ± 13.5% for MVR and MVP, respectively (*P* = 0.648). MVR showed a lower incidence of infective endocarditis relapse compared with MVP (MVR: 93.6 ± 7.1 versus MVP: 80.9 ± 10.8%, *P* = 0.041). At Cox regression, infective endocarditis relapse was an independent risk factor for death (hazard ratio 4.03; 95% confidence interval 1.41–11.52; *P* = 0.009).

**Conclusion:**

The tendency to postpone surgery in stable patients with mitral infective endocarditis allowed achievement of MVR in almost 50% of patients. Although repair remains the approach of choice in our institution, no differences between MVR and MVP were reported in terms of early/late survival. However, MVP had a higher incidence of infective endocarditis relapse that represents an independent risk of mortality.

## Introduction

Benefits of reparative strategies have been largely established for the surgical treatment of degenerative mitral valve diseases.^1,2^ However, clinical advantages of repair over replacement strategies seem to be less pronounced when new onset of mitral regurgitation is the consequence of an acute infective endocarditis.

Despite several studies reporting a superior overall survival of mitral valve repair (MVR) techniques over replacement,^3–6^ this approach might be limited or not feasible when a large amount of mitral tissue needs to be resected or, even worse, is lacking, thus limiting the reparability or increasing the complexity of the repair. In this scenario, concerns have been raised about durability of the repair, especially in technically challenging cases. Moreover, complex repair may require prolonged cardiopulmonary bypass time that might be cumbersome in this subset of patients. Additionally, complex repair techniques are related to a higher need for reinterventions at mid to long term.^7^

Conversely, replacement by means of a prosthetic valve [mitral valve prosthesis (MVP)] is nowadays limited to poorer surgical candidates thus significantly influencing perioperative and short-term results. Of note, this approach should be discouraged in patients with infective endocarditis secondary to intravenous drug abuse to limit the amount of prosthetic material and the incidence of recurrences.^8^

In this retrospective single-center study, we sought to critically investigate clinical outcomes derived from our institutional approach to patients with severe mitral valve regurgitation secondary to active infective endocarditis. We focused our analysis on the effects of repair or replacement at long-term follow-up.

## Methods

### Patients’ selection

We included all patients who underwent mitral valve surgery, either repair or replacement, because of active infective endocarditis on native mitral valve between January 2004 and December 2019. The Institutional Review Board approved this study (PN 1815). According to the ESC criteria, active infective endocarditis was defined as surgery performed within 6 weeks of antibiotic treatment onset, in the presence of positive culture of the remnants of tissue retrieved during surgery as well as macroscopic evidence of endocarditis. We therefore included patients with age older than 18 years with diagnosis of active infective endocarditis primarily affecting the mitral valve. Patients with previous mitral valve surgery were excluded from the current analysis.^9^

Finally, our study population consisted of 109 patients undergoing surgery for acute infective endocarditis on the native mitral valve. Of those, 53 patients (48.6%) underwent MVR whereas 56 patients (51.4%) had MVP.

Preoperative, intraoperative and postoperative data were retrieved from the institutional electronic medical records, while clinical follow-up consisted of routine postoperative outpatient visits as well as data collection by means of telephone interviews with patients and/or referral cardiologists. No patients were lost to follow-up.

### Preoperative management

Empiric antibiotic therapy was promptly initiated once diagnosis of infective endocarditis was confirmed according to the modified Duke criteria. Therapy was, therefore, tailored once the causative pathogen had been identified. Surgical intervention was considered and discussed case by case following the Heart Team decision involving anesthesiologists, cardiac surgeons, cardiologists and infectivologists. Furthermore, according to the ESC guidelines,^9^ surgery was deemed urgent in cases of uncontrolled infections, high risk of major embolism and in case of hemodynamic impairment because of infective endocarditis-related structural complications conditioning cardiac heart failure. Conversely, when urgency/emergency criteria were not met, surgery was postponed after completion of at least 4 weeks of antibiotic therapy. The goal of this approach was to raise the probability of blood as well as tissue sterilization, minimizing the risk of acute re-infection.

All operations were performed by experienced surgeons with extensive expertise in mitral valve repair and replacement.

According to Carpentier's general principle, the aims of the MVR were to restore a proper leaflet motion and coaptation and stabilize to annulus whenever possible.^10^ Thus, the first-line strategy in any surgical intervention was to assess the mitral valve reparability and proceed to MVR. After extensive removal of pathological or infected tissue, replacement was performed whenever the above general principles were deemed not achievable.

### Statistical analysis

Continuous variables are expressed as mean ± standard deviation and categorical variables as numbers (percentages). Continuous variables were compared by using Student's *t*-test or the Mann–Whitney–Wilcoxon test, as appropriate, whereas categorical variables were compared by using Pearson's *χ*^2^ test or Fisher's exact test, as appropriate. *P*-values of 0.05 or less were considered statistically significant.

Overall survival as defined above was estimated using the Kaplan–Meier method and survival curves were compared using the Mantel–Cox log-rank test. A Cox regression model was applied in order to identify predictors for mortality. The significant level *α* = 0.2 in the univariate analysis was used to select variables for the multivariate model (using backward stepwise elimination).

Primary end points were perioperative mortality and overall survival at 10 years of follow-up. Secondary end point was the event-free survival intended as survival free from endocarditis.

## Results

### Preoperative

Patient baseline characteristics are depicted in Table 1. No differences have been reported among the two groups and the Operative Risk Evaluation (EuroSCORE II). LVEF values were comparable despite the tendency in the MVR group to have a higher incidence of male patients and intravenous drug abusers. However, this did not reach statistical relevance. A total of 22 (20.2%) patients had had previous cardiac surgery: 9 had had previous aortic valve replacement whereas 13 had had previous coronary artery bypass grafting (CABG).

**Table 1:** Preoperative patient characteristics

Variable	Overall population (*n* = 109)	Mitral valve repair (MVR) (*n* = 53)	Mitral valve replacement (MVP) (*n* = 56)	*P*-value
Age (years) (mean ± SD)	58.9 ± 15.5	57.7 ± 15.7	60 ± 15.2	0.437
Male [*n* (%)]	74 (67.9%)	40 (75.5%)	34 (60.7%)	0.099
Hypertension [*n* (%)]	62 (56.9%)	30 (56.6%)	32 (57.1%)	0.955
S-creatinine (mg/dl) (mean ± SD)	1.45 ± 1.35	1.29 ± 1.09	1.72 ± 2.05	0.205
Atrial fibrillation [*n* (%)]	24 (22%)	10 (18.9%)	14 (25%)	0.440
BSA (m^2^) (mean ± SD)	1.77 ± 0.11	1.77 ± 0.11	1.78 ± 0.10	0.907
BMI (kg/m^2^) (mean ± SD)	23.78 ± 2.6	23.8 ± 2.7	23.7 ± 2.6	0.847
Smoker [*n* (%)]	32 (29.4%)	18 (34%)	14 (25%)	0.304
LVEF (%) (mean ± SD)	56.74 ± 9.1	56.79 ± 8.3	56.7 ± 9.83	0.956
NYHA 4 [*n* (%)]	18 (17.6%)	10 (19.2%)	8 (16%)	0.669
EuroSCORE II (mean ± SD)	7.27 ± 9.35	6.7 ± 8.31	8.15 ± 10.88	0.542
Severe mitral regurgitation [*n* (%)]	97 (89%)	47 (88.7%)	50 (89.3%)	0.920
Dialysis [*n* (%)]	3 (2.7%)	1 (1.9%)	2 (3.6%)	1
Diabetes with insulin (%)	13 (11.9%)	6 (11.3%)	7 (12.5%)	0.849
Previous stroke [*n* (%)]	24 (22%)	13 (24.5%)	11 (19.6%)	0.538
Hypercholesterolemia	39 (35.8%)	23 (43.4%)	16 (28.6%)	0.107
Previous cardiac surgery [*n* (%)]	22 (20%)	11 (20.8%)	11 (19.6%)	0.885

BSA, body surface area; LVEF, left ventricular ejection fraction; MVP, mitral valve prosthesis; MVR, mitral valve repair; NYHA, New York Heart Association; S-creatinine, serum creatinine; SD, standard deviation.

### Causative microorganism

Causative microorganisms are summarized in Table 2. The most frequently isolated pathogens were *Staphylococcus aureus* (17,4%), *Streptococcus viridans* (12.8%) and *Enterococcus* (10.1%) whereas in 34 cases (31.1%), blood cultures resulted as negative.

**Table 2:** Causative microorganisms

Pathogen	Total (*n* = 109)	Percentage
*Staphylococcus* spp.	27	24.8%
*Staphylococcus aureus*	19	17.4%
MSSA	11	10.1%
MRSA	8	7.3%
CNS (coagulase-negative *Staphylococcus*)	8	7.3%
*Staphylococcus epidermidis*	4	3.7%
*Staphylococcus haemoliticus*	1	0.9%
*Staphylococcus lugdudensis*	2	1.8%
*Staphylococcus capitis*	1	0.9%
*Streptococcus* spp.	28	25.7%
*Streptococcus viridans*	14	12.8%
*Streptococcus sanguinis*	8	7.3%
*Streptococcus mitis*	4	3.7%
*Streptococcus anginosus*	1	0.9%
*Streptococcus defectivus*	1	0.9%
*Streptococcus gallolyticus*	9	8.3%
Former nutritional variants	2	1.8%
*Abiotrofia defective*	1	0.9%
*Granulicatella adiacens*	1	0.9%
Other Streptococcaceae	3	2.8%
*Enterococcus faecalis*	11	10.1%
*Actinomyces odontolyiticus*	2	1.8%
HACEK group	2	1.8%
Gram negative other than HACEK	3	2.8%
*Candida* spp.	2	1.8%
Negative blood culture	34	31.1%

MRSA, methicillin-resistant *Staphylococcus aureus*; MSSA, methicillin-sensitive *Staphylococcus aureus*.

### Surgical technique

Surgical details are listed in Table 3. In 14 cases (12.8%), surgery was performed under urgency/emergency conditions because of hemodynamic instability (MVR 5 patients, 9.4%; MVP 9 patients, 16.1%, *P* = 0.301). Twenty-five patients (22.9%) underwent concomitant aortic valve replacement and five patients had concomitant CABG (4.6%). Valve replacement in the MVP group was performed via median sternotomy in all cases: 71.4% (40/56) of patients received a mechanical valve. Of note, posterior subvalvular apparatus was preserved unless largely involved in the infective endocarditis process. Conversely, median sternotomy was performed in 77.4% of patients of the MVR group whereas others underwent MVR via right anterior mini-thoracotomy (12/53, 22.6% patients). In particular, the MVR group required valvuloplasty associated with annuloplasty in 86.8% of cases (46/53), whereas a repair without annular stabilization was performed in 13.2% of patients (7/53). Technical details of MVR are reported in Table 4.

**Table 3:** Operative outcomes

Variables	Total (*n* = 109)	Mitral valve repair (MVR) (*n* = 53)	Mitral valve replacement (MVP) (*n* = 56)	*P*-value
Urgent/emergency surgery [*n* (%)]	14 (12.8%)	5 (9.4%)	9 (16.1%)	0.301
Concomitant aortic valve replacement [*n* (%)]	25 (22.9%)	14 (26.4%)	11 (19.6%)	0.401
Concomitant CABG [*n* (%)]	5 (4.6%)	4 (7.6%)	1 (1.8%)	0.198
CPB time (min), median (25th–75th IQR)	113 (95–13)	125 (104–143)	83 (80–96)	0.006
Aortic cross-clamp time (min), median (25th–75th IQR)	186 (80–102)	90 (75–106)	83 (80–96)	0.238

CABG, coronary artery bypass grafting; CPB, cardiopulmonary bypass; IQR, interquartile range; MVP, mitral valve prosthesis; MVR, mitral valve repair.

**Table 4:** Surgical techniques^a^ used for mitral valve repair

Surgical technique^a^ for MVR	Frequency
Edge to edge	10 (18.9%)
Plicature	2 (3.8%)
Commissural closure	2 (3.8%)
Leaflet extension	6 (11.3%)
P2 resection	25 (47.2%)
Other segments resection	3 (5.7%)
Gore-Tex chordal replacement	7 (13.2%)
Prosthetic ring	46 (86.8%)
Repair without ring	7 (13.2%)

MVR, mitral valve repair.

a Techniques described are used alone or combined.

### Perioperative

In-hospital mortality was 2.8% (3/109 patients). Of these, one patient in the MVR group died from cardiogenic shock whereas in the MVP group, two patients died from multiorgan failure and invasive fungal infection, respectively. Postoperative course and complications are reported in Table 5. Of note, no patients experienced cerebrovascular accidents during hospitalization. Mean hospital stay was 10.5 ± 9.10 days (MVR: 11.04  ± 10.4 days versus MVP: 10.07 ± 8.09 days, *P* = 0.580). During this time frame, no patients experienced early infective endocarditis relapse.

**Table 5:** Postoperative outcomes

Variables	Overall population (*n* = 109)	Mitral valve repair (MVR) (*n* = 53)	Mitral valve replacement (MVP) (*n* = 56)	*P*-value
In-hospital mortality [*n* (%)]	3 (2.8%)	1 (1.9%)	2 (3.6)	1
Length of stay (days) (mean ± SD)	10.5 ± 9.10	11.04 ± 10.4	10.07 ± 8.09	0.580
ICU stay (h) (median, IQR)	22 (18–54)	24 (18–66)	21 (18–42)	0.449
Prolonged ventilation (MAV >48 h)	5 (4.6%)	4 (7.5%)	1 (1.8%)	0.190
IABP [*n* (%)]	5 (4.6%)	3 (5.7%)	2 (3.6%)	0.602
Bleeding requiring surgical revision [*n* (%)]	6 (5.5%)	2 (3.8%)	4 (7.1%)	0.679
Blood transfusion [*n* (%)]	62 (56.9%)	36 (67.9%)	26 (46.4%)	**0.024**
Acute renal injury (KDIGO ≥2) [*n* (%)]	6 (5.5%)	4 (7.5%)	2 (3.6%)	0.429
Postoperative LVEF (mean ± SD)	55.06 ± 8.38	55.91 ± 8.05	54.16 ± 8.76	0.399

IABP, intra-aortic balloon pump; IQR, interquartile range; LVEF, left ventricular ejection fraction; MAV, mechanical assisted ventilation; MVP, mitral valve prosthesis; MVR, mitral valve repair; SD, standard deviation.

### Follow-up

**Fig. 1:**
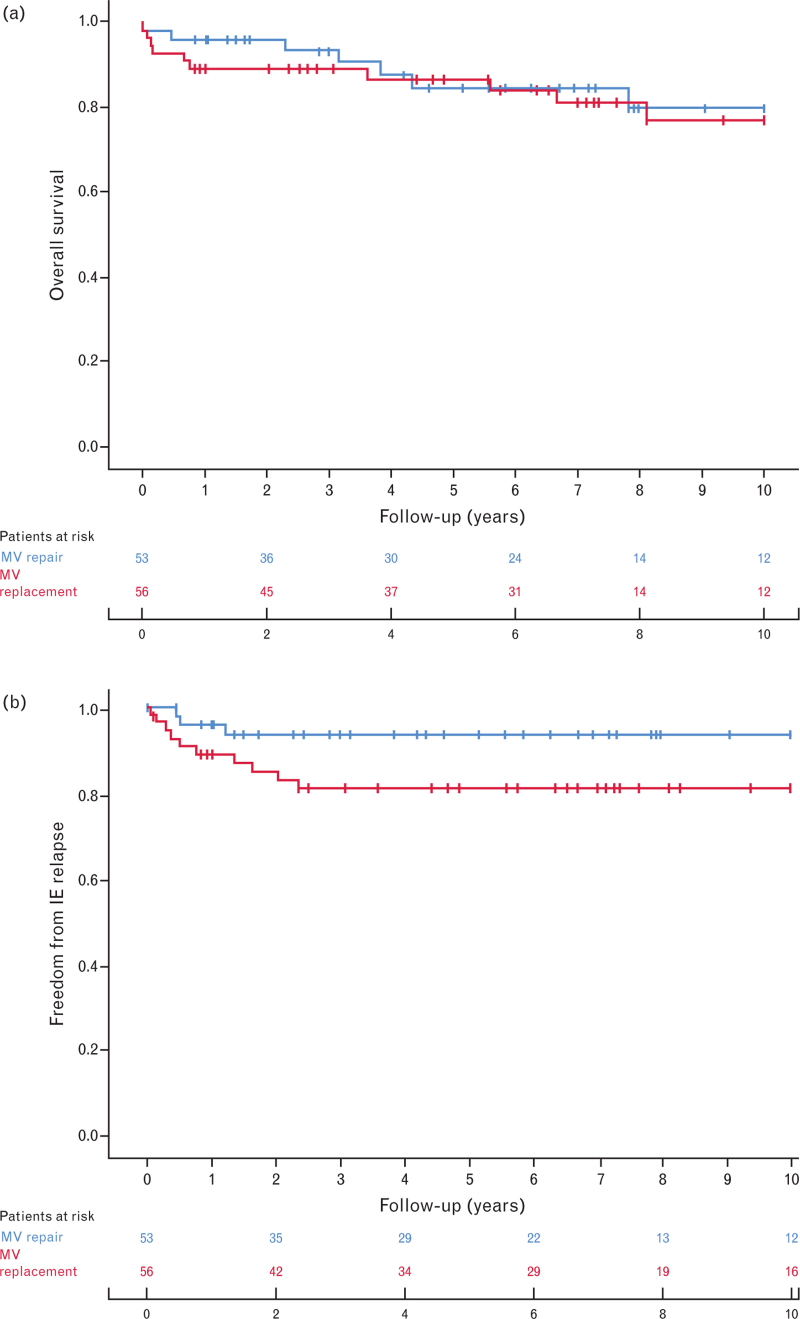
(a) Survival analysis with Kaplan–Meier curve for overall survival. (b) Kaplan–Meier curve for survival freedom from endocarditis relapse.

Mean follow-up was 74 months [95% confidence interval (CI) 23–126] and the follow-up time was comparable between the two groups (MVP: 69 ± 52 months versus MVP: 78 ± 52 months, *P* = 0.294). All-cause mortality rate was 15.6% (17/109) for the entire population and no differences were found between the two groups (MVR 7/53, 13.2% versus MVP 10/56, 17.9%, *P* = 0.503). Survival rate at 5 years of follow-up was 84.7 ± 5.1% for MVR and 86.9 ± 9.0% for MVP whereas at 10-year follow-up, survival was 80.0 ± 14.1 and 77.2 ± 13.5% for MVR and MVP, respectively (*P* = 0.648) (Fig. 1a). Cardiac-related death occurred in three MVR and five MVP patients (*P* = 0.717).

Infective endocarditis relapse was confirmed in 13 patients [MVR = 3/53 (5.7%) versus MVP = 10/56 (16.1%), *P* = 0.049]; in particular, 3 of these required reintervention (1 in the MVR group and 2 in the MVP group). Kaplan–Meier event-free survival showed a significantly higher incidence of endocarditis relapse in the MVP group (10-year: MVR = 93.6 ± 7.1; MVP = 80.9 ± 10.8%, *P* = 0.041; Fig. 1b). Multivariate Cox regression analysis depicted endocarditis relapse as an independent predictor of mortality (hazard ratio = 4.03; 95% CI 1.41–11.52; *P* = 0.009). Cox regression analysis is reported in detail in Table 6.

**Table 6:** Univariable and multivariable Cox regression analysis to identify independent predictors of all-cause mortality at follow-up

	Univariable			Multivariable		
	HR	95% CI	*P*-value	HR	95% CI	*P*-value
MRSA/MSSA	1.11	0.60–2.04	0.737			
REDO	0.56	0.14–2.23	0.417			
Drug abuser	0.85	0.41–1.75	0.668			
MVR versus MVP	0.63	0.39–1.01	0.058	0.99	0.37–2.68	0.986
Early surgery	0.81	0.38–1.76	0.597			
IE recurrence	4.03	1.41–11.52	0.009	2.98	1.37–6.47	0.006

HR, hazard ratio; IE, infective endocarditis; MRSA, methicillin-resistant *Staphylococcus aureus*; MSSA, methicillin-sensitive *Staphylococcus aureus*; MVP, mitral valve prosthesis; MVR, mitral valve repair.

Eight redo operations were performed on mitral valve/mitral prosthesis during the follow-up: three for re-endocarditis as outlined above (one in the MVR group and two in the MVP group), three for worsening of mitral regurgitation after a previous MVR and two for prosthesis degeneration.

The incidence of cardiac-related need for rehospitalization was 29.4% (32/109) at long-term follow-up [MVR = 28.3% (15/53); MVP = 30.4% (17/56), *P* = 0.814].

## Discussion

This study sought to examine the clinical outcomes of patients undergoing MVR compared with MVP as treatment for acute infective endocarditis. One hundred and nine patients were enrolled from January 2014 to December 2019 where 48.6% underwent MVR and 51.4% of patients had MVP. We found no advantages in terms of overall survival reported at long-term follow-up when MVR was performed over MVP. However, MVP was associated with a higher incidence of infective endocarditis relapse, which was found to be an independent predictor of mortality.

The principle of endocarditis surgery is to completely eradicate infection and restore functional anatomy of the involved valve. Since its first report in the 1990s, MVR in infective endocarditis has gained popularity and it has been largely proposed as the treatment of choice.^11^ Nowadays, leading centers in mitral valve surgery reach 80% of repairs in infective endocarditis on mitral valve.^12^ However, in the ‘real world’ the median of MVR performed in infective endocarditis is around 15% with a wide variability, ranging from 10 to 80%.^12,13^

In this study, MVR was retained as feasible in 50% of patients with acute infective endocarditis when the mitral valve was involved in an infectious process. Unless the indications for urgent/emergent surgery were met according to the ESC guidelines, surgery was postponed after 4 weeks of antibiotic therapy.^9^ This approach aimed to achieve a full blood sterilization, maximizing medical therapy before surgery, lowering the incidence of acute infective endocarditis relapse and reducing complications in the postoperative course for either the MVR or the MVP group. Defauw *et al.*^14^ described a different timing for surgical intervention and showed early results akin to those in our study. In their study, surgery was performed within a few days of optimal antibiotics therapy to minimize progression of the disease, which resulted in no early postoperative reinfection. Similarly, de Kerchove *et al.*^12^ described an early surgical approach (within a median delay of 9 days – unless urgent) with mitral valve surgery being performed. They reported only one case of early relapse of infective endocarditis despite 4.6% of cases requiring early reintervention for suture dehiscence (four in the MVP group and one in the MVR group) thus suggesting that early surgery performed on active inflammatory tissue may jeopardize the effectiveness of the surgical procedure. However, both centers reported an intraoperative and perioperative mortality of around 15%, respectively. This was five-fold higher than what we found in our population (2.8%; 3/109) following a maximization of medical therapy reducing the risk of late bacteremia. These differences in early results keep topic controversies between pros and cons of an early versus a delayed surgical approach. Moreover, de Kerchove e*t al.*,^12^ Cuerpo *et al.*^15^ and Ruttmann *et al.*^16^ refuted the general idea of ‘urgent surgery reduces itself the repair probability on the mitral valve’ being able to proceed to MVR in more than 60 and 80% of cases.

We speculate a watchful waiting approach in nonurgent/emergency clinical scenarios that allows the infective endocarditis process to expand and reduce healthy tissue available for a stable and durable repair. The appropriate timing for the treatment of infective endocarditis involving ‘left-side valves’ is still a matter of debate and results in literature showed to be inconclusive in demonstrating a clear superiority of a ‘prompt’ rather than a ‘delayed’ approach.

Our population was balanced in terms of preoperative baseline characteristics. We did not outline any survival benefit of having the mitral valve repaired rather than replaced (MVR: 80.0 ± 14.1% versus MVP: 77.2 ± 13.5%, *P* = 0.648). This was similar to results retrieved from the Spanish nationwide registry in which the tendency to have a better survival in the MVR group did not reach statistical significance.^15^ Other studies showed similar findings with no differences in terms of long-term survival in patients receiving MVR when compared with MVP.^17^ In contrast, a large US registry involving more than 1900 patients reported a significantly higher perioperative mortality at long-term follow-up in patients with infective endocarditis who underwent MVP when compared with MVR.^18^ Other groups reported similar findings where MVR showed survival benefits, especially for composite end points, including infective endocarditis relapse, mortality and need for redo surgery.^16^

Regarding the recurrences of infective endocarditis, we found a significantly higher incidence of relapse at 10-year follow-up in the MVP group compared with the MVR group (MVR: 3/53, 5.7% versus MVP: 10/56, 16.1%, *P* = 0.049). Most of these events occurred within the first 3 years of follow-up. These outcomes were confirmed in a recent meta-analysis by He *et al.*,^13^ where MVR was associated with a lower incidence of recurrences, thus suggesting that the prosthetic valve itself represents a plausible risk factor for endocarditis relapse.^18,19^ Opposite results were reported by Cuerpo *et al.*,^15^ along with Ruttmann *et al.*^16^ and Defauw *et al.*,^14^ where no differences were found in terms of infective endocarditis relapse. Despite controversies, in this study, we highlighted infective endocarditis as an independent predictor of mortality regardless of the surgical strategy (hazard ratio 4.03; 95% CI 1.41–11.52; *P* = 0.009).

### Limitations

The main limitation is the retrospective form of this current analysis. Despite the fact that we only included patients with evidence of mitral valve endocarditis involvement receiving MVR or MVP with/without associated procedures, the presence of patients who underwent urgent surgery for hemodynamic instability could have influenced early results in terms of morbidity and mortality. Although the preoperative variables were not statistically different, confounding factors may persist given the different surgical strategies chosen (MVR/MVP) and indicating the different mitral valve involvement between the two groups. Finally, the small sample size of this single-center report and lack of randomization limit the generalization of our findings.

## Conclusion

In conclusion, controversial data have been reported for early and late mortality and infective endocarditis relapse in patients undergoing MVR or MVP for infective endocarditis affecting the mitral valve. In this regard, a clear recommendation of a repair rather than replacement approach seems to be inappropriate. We do believe the therapeutic approach should be based on the individual circumstances as well as on the single-center results and experience. Further investigations are warranted, although powered randomized analyses comparing MVR versus MVP are difficult to perform in the context of infective endocarditis and they might be unethical.

## Acknowledgements

Data availability statement: The data underlying this article will be shared on reasonable request to the corresponding author.

IRB Approval PN 1815.

### Conflicts of interest

There are no conflicts of interest.

## References

[1] Jung JC Jang MJ Hwang HY. Meta-analysis comparing mitral valve repair versus replacement for degenerative mitral regurgitation across all ages. *Am J Cardiol* 2019; 123:446–453. 30471709 10.1016/j.amjcard.2018.10.024

[2] Gillinov AM Blackstone EH Nowicki ER. Valve repair versus valve replacement for degenerative mitral valve disease. *J Thorac Cardiovasc Surg* 2008; 135:885–893. 893.e1–893.e2. 18374775 10.1016/j.jtcvs.2007.11.039

[3] Okada Y Nakai T Muro T Ito H Shomura Y. Mitral valve repair for infective endocarditis: Kobe experience. *Asian Cardiovasc Thorac Ann* 2020; 28:384–389. 32757655 10.1177/0218492320947586 PMC7818674

[4] Tepsuwan T Rimsukcharoenchai C Tantraworasin A. Comparison between mitral valve repair and replacement in active infective endocarditis. *Gen Thorac Cardiovasc Surg* 2019; 67:1030–1037. 31049817 10.1007/s11748-019-01132-4

[5] Lee HA Cheng YT Wu VC. Nationwide cohort study of mitral valve repair versus replacement for infective endocarditis. *J Thorac Cardiovasc Surg* 2018; 156:1473–1483. 29843917 10.1016/j.jtcvs.2018.04.064

[6] Harky A Hof A Garner M Froghi S Bashir M. Mitral valve repair or replacement in native valve endocarditis? Systematic review and meta-analysis. *J Card Surg* 2018; 33:364–371. 29926515 10.1111/jocs.13728

[7] Liu X Miao Q Liu X Zhang C Ma G Liu J. Repair versus replacement for active endocarditis of the mitral valve: 9 years of experience. *J Card Surg* 2022; 37:3713–3719. 36073065 10.1111/jocs.16904

[8] Cecchi E Corcione S Lupia T. Infective endocarditis in people who inject drugs: report from the Italian Registry of Infective Endocarditis. *J Clin Med* 2022; 11:4082. 35887843 10.3390/jcm11144082 PMC9319987

[9] Habib G Lancellotti P Antunes MJ. 2015 ESC Guidelines for the management of infective endocarditis: the Task Force for the Management of Infective Endocarditis of the European Society of Cardiology (ESC). Endorsed by: European Association for Cardio-Thoracic Surgery (EACTS), the European Association of Nuclear Medicine (EANM). *Eur Heart J* 2015; 36:3075–3128. 26320109 10.1093/eurheartj/ehv319

[10] Carpentier A Adams DH Filsoufi F. Carpentier's reconstructive valve surgery: from valve analysis to valve reconstruction. Maryland Heights, Missouri: Saunders/Elsevier; 2010.

[11] Dreyfus G Serraf A Jebara VA. Valve repair in acute endocarditis. *Ann Thorac Surg* 1990; 49:706–711. 2339926 10.1016/0003-4975(90)90007-s

[12] de Kerchove L Price J Tamer S. Extending the scope of mitral valve repair in active endocarditis. *J Thorac Cardiovasc Surg* 2012; 143: (4 Suppl): S91–S95. 22306214 10.1016/j.jtcvs.2012.01.049

[13] He K Song J Luo H. Valve replacement or repair in native mitral valve infective endocarditis-which is better? A meta-analysis and systematic review. *J Card Surg* 2022; 37:1004–1015. 35032059 10.1111/jocs.16227

[14] Defauw RJ Tomšič A van Brakel TJ Marsan NA Klautz RJM Palmen M. A structured approach to native mitral valve infective endocarditis: is repair better than replacement? *Eur J Cardiothorac Surg* 2020; 58:544–550. 32333009 10.1093/ejcts/ezaa079 PMC7453034

[15] Cuerpo GP Valerio M Pedraz A. Mitral valve repair in infective endocarditis is not inferior to valve replacement: results from a Spanish nationwide prospective registry. *Gen Thorac Cardiovasc Surg* 2019; 67:585–593. 30666586 10.1007/s11748-019-01063-0

[16] Ruttmann E Legit C Poelzl G. Mitral valve repair provides improved outcome over replacement in active infective endocarditis. *J Thorac Cardiovasc Surg* 2005; 130:765–771. 16153926 10.1016/j.jtcvs.2005.03.016

[17] Perrotta S Fröjd V Lepore V Schersten H Jeppsson A Svensson G. Surgical treatment for isolated mitral valve endocarditis: a 16-year single-centre experience. *Eur J Cardio-thorac Surg* 2018; 53:576–581. 10.1093/ejcts/ezx416 29186533

[18] Toyoda N Itagaki S Egorova NN. Real-world outcomes of surgery for native mitral valve endocarditis. *J Thorac Cardiovasc Surg* 2017; 154:1906.e9–1912.e9. 28942975 10.1016/j.jtcvs.2017.07.077

[19] Oliver L Leauthier M Jamme M. Mitral valve repair is better than mitral valve replacement in native mitral valve endocarditis: results from a prospective matched cohort. *Arch Cardiovasc Dis* 2022; 115:160–168. 35249849 10.1016/j.acvd.2022.02.002

